# A non-randomized, open-label, single-arm, Phase 2 study of emibetuzumab in Asian patients with MET diagnostic positive, advanced gastric cancer

**DOI:** 10.1007/s00280-017-3445-z

**Published:** 2017-10-25

**Authors:** Daisuke Sakai, Hyun Cheol Chung, Do-Youn Oh, Se Hoon Park, Shigenori Kadowaki, Yeul Hong Kim, Akihito Tsuji, Yoshito Komatsu, Yoon-Koo Kang, Kazunori Uenaka, Sameera R. Wijayawardana, Volker Wacheck, Xuejing Wang, Ayuko Yamamura, Toshihiko Doi

**Affiliations:** 1 Osaka University Hospital, Osaka, 565-0871 Japan; 2 Yonsei Cancer Center, Yonsei University College of Medicine, Seoul, 03722 South Korea; 3 Seoul National University Hospital, Seoul, 03080 South Korea; 4 Cancer Research Institute, Seoul National University College of Medicine, Seoul, 03080 South Korea; 5 Sungkyunkwan University Samsung Medical Center, Seoul, 06351 South Korea; 6 Aichi Cancer Center Hospital, Nagoya, 464-8681 Japan; 7 Korea University College of Medicine, Seoul, 02841 South Korea; 8 Department of Clinical Oncology, Faculty of Medicine, Kagawa University, Kita, 761-0793 Japan; 9 Cancer Center, Hokkaido University Hospital, Sapporo, 060-8648 Japan; 10 Asan Medical Center, University of Ulsan College of Medicine, Seoul, 138-736 South Korea; 11 Eli Lilly Japan K.K., Kobe, 651-0086 Japan; 12 Eli Lilly and Company, Indianapolis, IN 46285 USA; 13 National Cancer Center Hospital East, 5-1, Kashiwanoha 6-chome, Kashiwa, Chiba 277-8577 Japan

**Keywords:** Antibodies, monoclonal, humanized, Clinical trial, Phase II, LY2875358, MET protein, human, Stomach neoplasms

## Abstract

**Purpose:**

Mesenchymal–epithelial transition factor (MET) is expressed in gastric cancer and associated with poor clinical outcomes. We assessed activity, safety, and pharmacokinetics of emibetuzumab, a bivalent monoclonal anti-MET antibody that blocks ligand-dependent and ligand-independent MET signaling.

**Methods:**

This non-randomized, single-arm, Phase 2 study enrolled Asian patients with MET diagnostic positive advanced gastric adenocarcinoma. Emibetuzumab (2000 mg, intravenous) was given on days 1 and 15 (28-day cycle). The primary endpoint was 8-week progression-free survival rate. Secondary objectives included safety, pharmacokinetics, overall survival, and change in tumor size.

**Results:**

Tumors from 65 patients were immunohistochemically screened to enroll 15 MET diagnostic positive patients (23% positivity; 8 Japanese, 7 Korean; 10 male). Eight-week progression-free survival rate was 0.47 (70% CI, 0.33–0.59). Disease control rate was 40% (target lesion decreases, three patients; no complete/partial responses according to RECIST). Median overall survival was 17.1 weeks (95% CI, 6.3–not achievable). No serious emibetuzumab-related adverse events or new safety signals emerged. Grade ≥ 3 possibly drug-related adverse events were hyperkalemia, hyponatremia, and hyperuricemia (one each). Emibetuzumab’s pharmacokinetics profile was similar to that observed previously. MET expression and clinical outcomes were not obviously associated.

**Conclusion:**

Emibetuzumab was well tolerated with limited single-agent activity in advanced gastric adenocarcinoma.

## Introduction

The mesenchymal–epithelial transition factor (MET) receptor is a tyrosine kinase receptor and its only known ligand is hepatocyte growth factor (HGF). HGF/MET signaling is involved in embryogenesis and responses to tissue damage, and MET is broadly expressed in normal adult tissues [1]. Activation of MET signaling can occur via a ligand-dependent mechanism, by HGF, or ligand-independent mechanisms, including overexpression of the MET protein, amplification of the MET gene (*c-met*), or *c-met* mutations [1, 2]. Aberrant MET signaling has been described to be involved in tumor growth and invasion, angiogenesis, metastasis, and resistance to therapy [1–3]. MET expression has been reported in many tumor types, including gastric cancer [3]. It has been suggested that *c-met* amplification and overexpression of MET protein in gastric cancer correlate with poor patient outcomes [4]. Clinical studies with MET-targeting agents have demonstrated a role for MET as a predictive biomarker in patients with gastric cancer [5].

Emibetuzumab is a humanized immunoglobulin G4 monoclonal bivalent anti-MET antibody that blocks MET signaling via two distinct mechanisms: it suppresses ligand-dependent MET activation by blocking HGF interaction with the receptor, and it suppresses ligand-independent MET activation by causing the MET receptor to be internalized and degraded [6]. Pre-clinical research has shown that emibetuzumab inhibits MET-expressing gastric cancer cell line proliferation in vitro and in vivo when given as monotherapy or in combination with chemotherapy [7]. In Phase 1 dose escalation studies, emibetuzumab monotherapy was well tolerated in patients with advanced solid tumors, including gastric cancer, with no dose-limiting toxicities (Studies JTBA and JTBD) [8, 9]. Emibetuzumab activity in patients with advanced gastric cancer has not previously been evaluated, and there is limited information on biomarkers that might help identify patients with gastric cancer who will respond to emibetuzumab treatment.

The aim of this Phase 2 study was to explore the antitumor activity, safety, and pharmacokinetics of emibetuzumab in patients with advanced gastric or gastroesophageal junction (GEJ) adenocarcinoma selected for positive MET tumor expression (MET diagnostic positive). The primary objective of the study was to evaluate emibetuzumab activity in terms of 8-week progression-free survival (PFS) rate relative to historical control.

## Materials and methods

### Study design

This study was a non-randomized, open-label, single-arm, Phase 2 study of emibetuzumab in Japanese and Korean patients with MET diagnostic positive advanced gastric or GEJ adenocarcinoma conducted in 12 study centers between October 2013 and December 2014 (Study JTBE). The study was registered at http://www.clinicaltrials.gov (NCT01874938). The study protocol conformed to the Declaration of Helsinki and the International Conference of Harmonisation Good Clinical Practice guidelines, and was approved by the ethics review board at each study site. All patients provided written informed consent to provide a tissue sample for prescreening for diagnosis of MET expression status and to undergo study-specific procedures.

### Study population

Patients with a diagnosis of histopathologically or cytologically confirmed gastric or GEJ adenocarcinoma, who had locally advanced and/or metastatic disease that was unresectable, were ≥ 20 years, and had not previously been treated with any HGF-/MET-targeting therapeutics, were screened for eligibility for the study. As a prescreening step, patients’ tumor samples were tested for MET protein expression status by immunohistochemistry (IHC). Archival tumor tissues, or biopsy samples taken at prescreening, were tested for MET protein expression status by IHC at an Eli Lilly and Company central laboratory using the A2H2-3 diagnostic anti-MET antibody [10]. Patients were eligible if they were MET positive, which was defined as ≥ 60% of tumor cells staining at 2 + or 3 + intensity for MET.

Patients who were identified to be MET diagnostic positive were further screened for enrollment using the following inclusion criteria: two prior chemotherapy regimens containing fluoropyrimidine and platinum agents for gastric or GEJ adenocarcinoma; measurable disease as defined by the Response Evaluation Criteria in Solid Tumors (RECIST) version 1.1 [11]; adequate organ function; and performance status of ≤ 1 on the Eastern Cooperative Oncology Group scale. Exclusion criteria included: active fungal, bacterial, and/or known viral infection; heart failure classified New York Heart Association class ≥ 3, unstable angina, or myocardial infarction in the previous 6 months; and a corrected QT interval of > 470 ms on the screening electrocardiogram.

### Study treatment

Patients received emibetuzumab 2000 mg using a flat dosing scheme, by intravenous infusion over 150 min on day 1 and day 15 of a 28-day cycle. Treatment was continued until at least one discontinuation criterion was met [eg, progressive disease (PD), unacceptable toxicity].

### Clinical assessments

Tumor response and progression were evaluated using RECIST version 1.1, and graded adverse events (AEs) using the National Cancer Institute Common Terminology Criteria for Adverse Events version 4.03. To determine serum concentrations of emibetuzumab, blood samples were collected before drug infusion and at the end of drug infusion on day 1 and day 15 during each of the first four cycles.

### Primary and secondary outcome measures

The primary efficacy measure of the study was the PFS rate at 8 weeks + 3 days. The timepoint for PFS rate was set at 8 weeks + 3 days, because tumor measurement at 8 weeks was done 3 days before or after 8 weeks, but it is referred to hereafter as 8 weeks. The secondary measures were: disease control rate (DCR), overall response rate, duration of response, overall survival (OS), PFS, best percentage change in tumor size, drug safety [assessed by AEs], and pharmacokinetic properties of the drug (serum concentrations at each sampling point).

### Exploratory biomarker analyses

For the exploratory biomarker analysis, MET protein expression in MET diagnostic positive patients was further categorized as high MET or low MET by different cut points for high expression. Levels of MET protein expression were assessed using a semi-quantitative scoring scheme that incorporated both the staining intensity and the percentage of cells displaying each level of staining intensity. These assessments were used to calculate the H-score for the cell membrane, as follows: *H*-score = 1 × (% of cells staining at 1 +) + 2 × (% of cells staining at 2 +) + 3 × (% of cells staining at 3 +) [12]. The cut points investigated for high MET or low MET among MET diagnostic positive patients were: > 30% of cells staining at 3 + intensity and *H*-score > 210 in the membrane; > 30% of cells staining at 3 + intensity in the membrane; *H*-score > 210 in the membrane; ≥ 80% of cells staining at ≥ 2 + intensity in the membrane; and 100% of cells staining at ≥ 2 + intensity in the membrane.

Fluorescence in situ hybridization (FISH) was used to assess *c-met* and epidermal growth factor receptor gene (*EGFR*) amplification of tumor samples using two different definitions of each. First, the number of cells with amplification was analyzed and tumor samples were considered amplified if ≥ 40% of the cells counted had ≥ 4 signals. Second, the ratio of cells with amplification was measured and tumor samples were considered amplified if the average number of signals divided by the average number of signals from the chromosome enumeration probe (CEP7) was ≥ 2.

### Sample size and statistical analyses

A sample size of 15 was determined, based on feasibility and the design of a previous Phase 1 study of emibetuzumab conducted in mostly Caucasian patients with advanced solid tumors (Study JTBA) [13]. This sample size provides a 78.6% power based on a one-sample test with one-sided type I error of 0.15 when the expected and threshold PFS rates at 8 weeks are assumed as 0.63 and 0.40, respectively.

Clinical activity and safety analyses were conducted for all patients who received at least one dose of emibetuzumab. For clinical activity parameters, point estimates and confidence intervals (CIs) were obtained. PFS rate was calculated using the Kaplan–Meier method. To address the primary objective, the lower limit of the two-sided 70% CI of the PFS rate at 8 weeks was compared with the threshold value of 0.40 to determine whether this limit was greater than the threshold value. For safety, AEs were summarized as the number and percentage of patients for whom each event was reported. SAS 9.2 (SAS Institute, Cary, NC, USA) was used for all statistical analyses.

## Results

### Patient disposition and baseline characteristics

In total, 65 patients from 12 study sites (7 in Japan, 5 in Korea) were prescreened for diagnosis of MET protein expression status. Of these patients, 22 were identified as being MET diagnostic positive (≥ 60% of tumor cells staining at 2 + or 3 + intensity for MET)—a MET diagnostic positive rate of 34% (22 of 65 patients). Seven of these 22 patients were not enrolled owing to patient decision (two patients), physician decision (two patients), sponsor decision (one patient, for whom prior therapy was ongoing and data on disease progression were incomplete at time of study enrollment), death due to study disease (one patient), or meeting the exclusion criterion of having an active fungal, bacterial, and/or known viral infection (one patient).

All 15 enrolled patients had an initial diagnosis of gastric adenocarcinoma; the majority were male, and the median patient age was 63 years (Table 1). All had received prior systemic therapy, with at least three prior therapies (range 3–5); six had received prior surgery, and one had received prior radiotherapy.

**Table 1:** Patient demographics and baseline characteristics

Variable	*N* = 15
Age, median (min–max) (years)	63 (39–74)
Male, *n* (%)	10 (67)
Country, *n* (%)	
Japan (three sites)	8 (53)
Korea (three sites)	7 (47)
ECOG performance status, *n* (%)	
0	1 (7)
1	14 (93)
Height, median (min–max) (cm)	164.5 (150.8–178.2)
Weight, median (min–max) (kg)	55.2 (39.5–75.6)
BMI, median (min–max) (kg/m^2^)	21.0 (17.2–25.4)
Pathological diagnosis at initial diagnosis, *n* (%)	
Gastric adenocarcinoma	15 (100)
GEJ adenocarcinoma	0
Prior therapies, *n* (%)	
Systemic therapy	15 (100)
Surgery	6 (40)
Radiotherapy	1 (7)
Number of measurable lesions (target lesions), *n* (%)^a^	
1	2 (13)
2	8 (53)
3	4 (27)
4	1 (7)
Number of metastatic lesions (non-gastric target lesions), *n* (%)^b^	
1	3 (20)
2	7 (47)
3	4 (27)
4	1 (7)
Number of metastatic sites (non-gastric target lesions), *n* (%)^c^	
1	9 (60)
2	4 (27)
3	1 (7)
4	1 (7)

*BMI* body mass index, *ECOG* Eastern Cooperative Oncology Group, *GEJ* gastroesophageal junction

^a^Target lesions that were present and measured at screening (detected by computed tomography)

^b^Non-gastric target lesions that were present and measured at screening (detected by computed tomography)

^c^Locations of non-gastric target lesions that were present and measured at screening (detected by computed tomography); locations were: liver, lymph node, ovary, peritoneum, rectouterine pouch, spleen, lung, colon, colorectal, bladder, periaortic

The median number of cycles of emibetuzumab given (one or both of the day 1 and day 15 doses administered) was 2 (range 1–10), and the median number of cycles completed (day 1 and day 15 doses both administered) was 2. Six patients completed one cycle, three completed two cycles, two completed three cycles, two completed five cycles, and one completed nine cycles; only one patient did not complete any cycles. The study treatment was discontinued for all 15 patients, either because of PD (14 patients) or death due to study disease (one patient). Six patients had post-discontinuation therapy; for five patients, this was systemic therapy, and for one patient, it was radiotherapy.

### Clinical activity

**Fig. 1:**
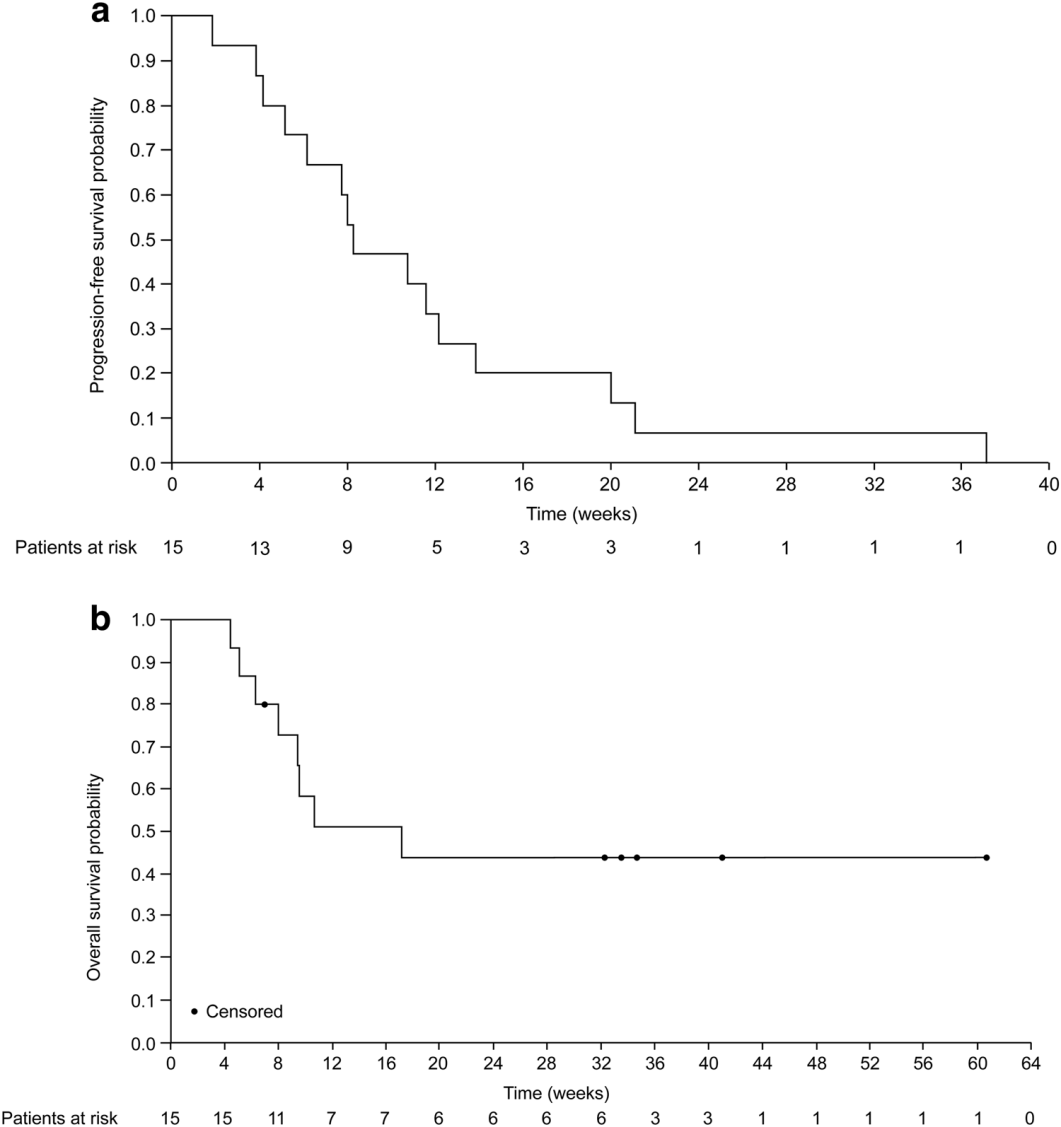
Kaplan–Meier analyses of progression-free survival (**a**) and overall survival (**b**)

The PFS rate at 8 weeks—the primary endpoint of this study—was 0.47 (70% CI, 0.33–0.59; Fig. 1a); the lower confidence limit did not exceed the prespecified threshold of 0.40. The median PFS was 8.3 weeks (95% CI, 4.14–12.14; Fig. 1a) and median OS was 17.1 weeks (95% CI, 6.3 to not achievable; Fig. 1b).

**Fig. 2:**
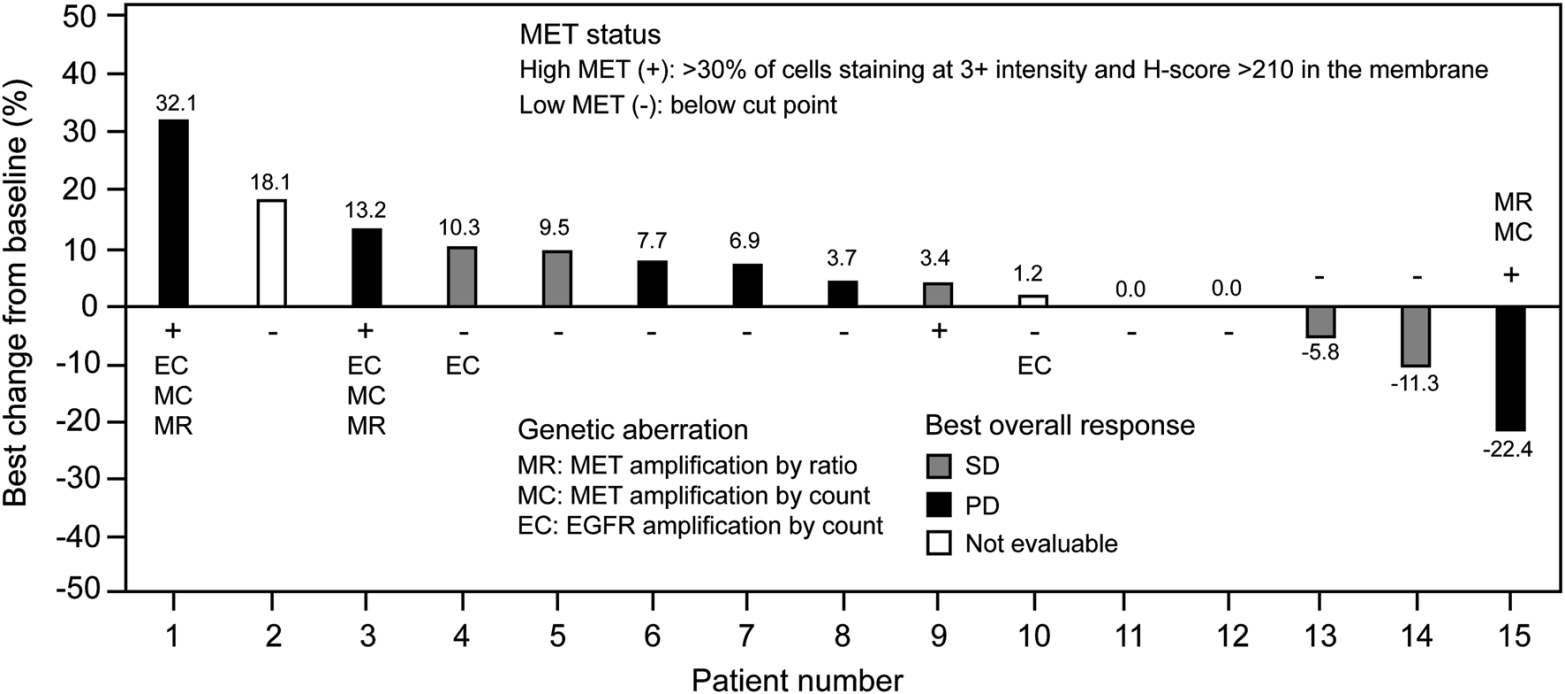
Waterfall plot showing percentage change in lesion size from baseline at best response and best overall response category for each patient. Tumors were considered not evaluable (according to RECIST) in patients with SD whose only post-baseline measurement occurred < 6 weeks after the first dose of emibetuzumab. The best overall response was SD for patient 11 and was not evaluable for patient 12. For patient 15, the best overall response was PD despite best change from baseline being − 22.4% because of a new lesion (malignant ascites). *EGFR* epidermal growth factor receptor, *MET* mesenchymal–epithelial transition factor, *PD* progressive disease, *RECIST* response evaluation criteria in solid tumors, *SD* stable disease

A total of six patients had a best overall response of stable disease during emibetuzumab monotherapy, resulting in a DCR of 40% (95% CI, 16–68%). No complete responses (CRs) or partial responses (PRs) according to RECIST were observed. The best percentage change in tumor size ranged from − 22.4 to 32.1%, and shrinkage in target lesions was observed in three patients (Fig. 2).

### Safety and tolerability measures

Emibetuzumab demonstrated a favorable safety profile and was well tolerated. Twelve patients (80%) experienced ≥ 1 AEs that were considered possibly study drug-related (Table 2). The most commonly reported possibly emibetuzumab-related AEs of any grade were constipation and hypoalbuminemia [three patients (20%) each; Table 2] and were all of mild or moderate severity. The only possibly emibetuzumab-related Grade ≥ 3 AEs reported were one case each of hyperkalemia (Grade 3), hyponatremia (Grade 3), and hyperuricemia (Grade 4) (the latter two AEs occurred in one patient).

**Table 2:** Overview of AEs (*N* = 15)

Category of AE	Patients with ≥ 1 AE *n* (%)	
	Any grade	Grade ≥ 3
Regardless of causality	14 (93)	8 (53)
Related to emibetuzumab	12 (80)	2 (13)
Constipation	3 (20)	0 (0)
Hypoalbuminemia	3 (20)	0 (0)
Diarrhea	2 (13)	0 (0)
Hyperkalemia	2 (13)	1 (7)
Hyperuricemia	2 (13)	1 (7)
Hypocalcemia	2 (13)	0 (0)
Hyponatremia	2 (13)	1 (7)
Insomnia	2 (13)	0 (0)
Nausea	2 (13)	0 (0)

*AE* adverse event

No AEs led to treatment discontinuation or death. Serious AEs were observed in six patients (40%), of which none were considered related to emibetuzumab. For three patients (20%), there was a dose delay due to an AE, including one AE that the investigator considered at least possibly related to emibetuzumab (hyperkalemia). An abnormal value of QTcF (Fridericia’s QT correction) exceeding 500 ms and an increase of QTcF > 60 ms from baseline was observed in two Japanese patients at a single timepoint (one pre-dose and one post-dose). Both prolongations were observed at only one visit and no coinciding clinical symptoms related to QTc prolongation were reported (i.e., no arrhythmia, fainting, and sudden death).

### Pharmacokinetics

**Fig. 3:**
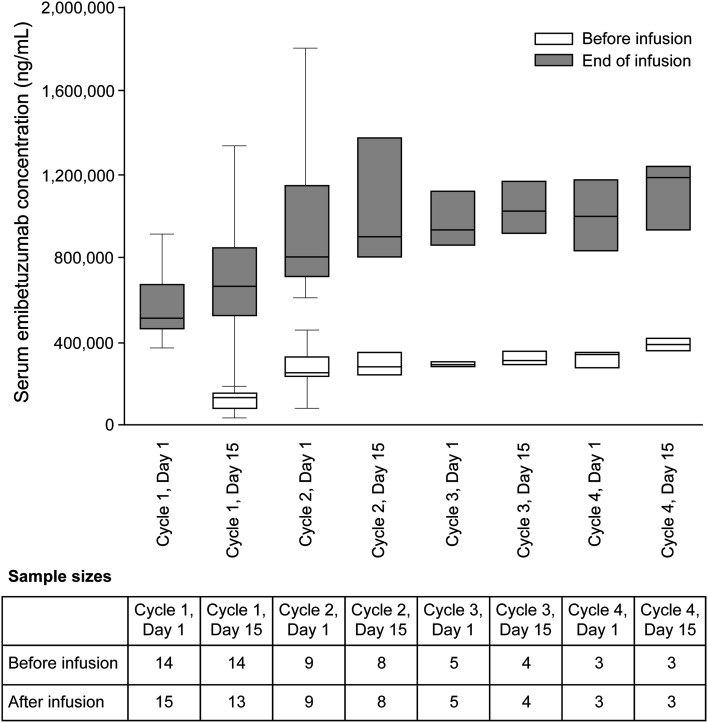
Serum concentrations of emibetuzumab before and after infusion of 2000-mg emibetuzumab during the first four cycles of therapy. A line within the box marks the median, and the boundaries of the box indicate the 75th and 25th percentiles. Whiskers above and below the box indicate the 90th and 10th percentiles

The serum concentration of emibetuzumab reached steady state in about 2–3 months (Fig. 3).

### Biomarker status and clinical outcomes

Tumor samples from nine patients were tested by both IHC and FISH. MET expression by IHC (using the threshold of > 30% of cells staining at 3 + intensity and *H*-score > 210 in the membrane) was categorized as high for four of these patients. Using FISH ratio and FISH count thresholds for *c-met* amplification (average number of *c-met* signals divided by average number of signals from CEP7 ≥ 2, and ≥ 40% of cells counted having ≥ 4 *c-met* signals, respectively), *c-met* was amplified for three of the four patients with high MET expression by IHC, and *c-met* was unamplified for all five of those with low MET expression by IHC. The association between high/low MET status and amplified/unamplified *c-met* status was significant, irrespective of whether the ratio or count definition was used for determining FISH amplification status (Fisher exact test, both *P* = 0.048).

**Fig. 4:**
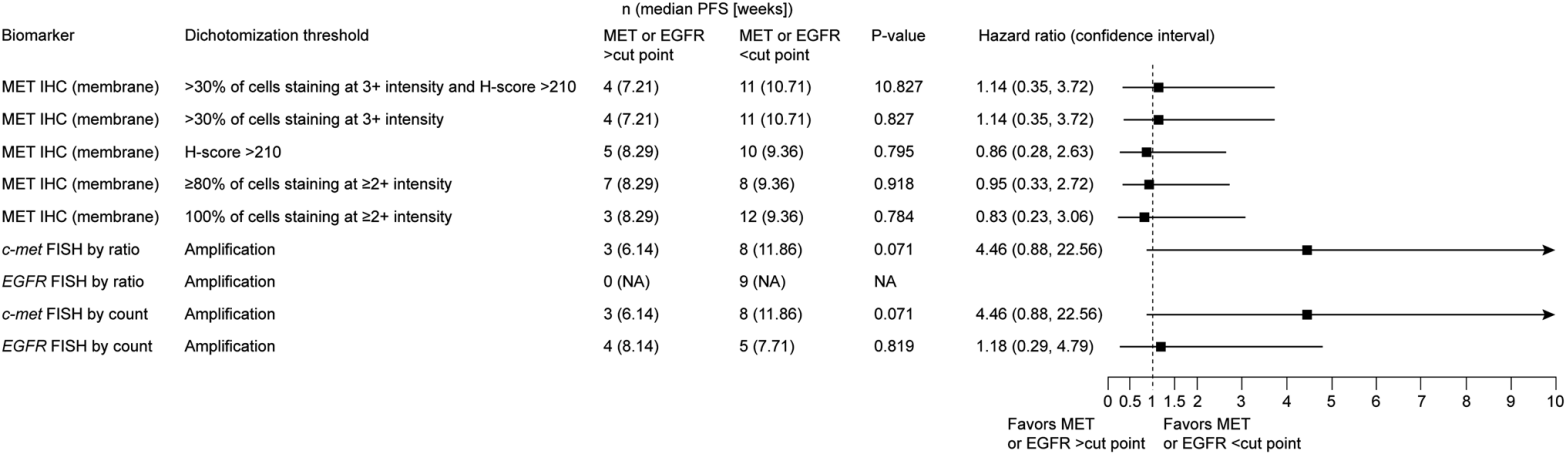
Forest plot of hazard ratios for progression-free survival by biomarker status. Abbreviations: *c-met* gene encoding MET, *EGFR* gene encoding epidermal growth factor receptor, *FISH* fluorescence in situ hybridization, *IHC* immunohistochemistry, *MET* mesenchymal–epithelial transition factor, *NA* not applicable, *PFS* progression-free survival

The small sample size of the study precluded the possibility of making a thorough assessment of the associations observed between biomarker status and clinical outcomes. Exploratory analysis did not show any difference in median PFS between patients classified as having high MET expression based on IHC and/or FISH using different cut points for high/low MET expression (Fig. 4).

*EGFR* amplification was observed in four out of nine patients’ tumor samples tested based on the cell count definition for amplification. Co-amplification of *c-met* and *EGFR* was observed in two out of these four tumor samples with *EGFR* amplification. None of the tumor samples showed *EGFR* focal amplification based on the ratio definition. No significant difference in median PFS was observed in patients with or without *c-met* or *EGFR* amplification by FISH ratio or FISH count.

## Discussion

This is the first study of emibetuzumab in patients with advanced gastric cancer selected for MET diagnostic positive status. Given as monotherapy to Asian patients with gastric cancer who had received two lines of prior cytotoxic therapy, emibetuzumab was well tolerated. However, even though the PFS rate at 8 weeks was 0.47, the study did not meet its prespecified primary endpoint as the lower confidence limit for this value did not exceed the threshold value of 0.40.

MET has been described as a negative prognostic marker for gastric cancer. In a recent meta-analysis that included data from more than 8000 patients with gastric cancer, IHC-determined MET overexpression was significantly correlated with aggressive tumor behavior [14]. In our study, all patients were MET diagnostic positive, which underscores the fact that the patient population selected for this trial should be considered as having an unfavorable prognosis. Still, 6 of 15 patients had a best overall response of stable disease, which corresponded to a DCR of 40%. The interpretation of this result is clearly limited by the absence of a control arm in our proof-of-concept study. However, it is possible that the aggressive tumor phenotype associated with MET expression might have contributed to the limited single-agent activity of emibetuzumab. Tumor shrinkage was observed in individual patients (three of 15 patients), but no CRs or PRs were observed for monotherapy in this study. Single-agent activity of MET-targeting agents has been described previously. In a Phase 2 study of patients with metastatic gastric cancer who received foretinib, an oral multikinase inhibitor that also targets MET, the best response was stable disease (achieved by 23 and 20% of patients who received intermittent dosing and daily dosing, respectively) [15]. In a Phase 2 study of patients with metastatic gastric cancer who received tivantinib, a selective inhibitor of *c-met*, no objective response was observed, and the DCR was 36.7% [16]. The reason for the limited single-agent activity of MET-targeting agents in gastric cancer remains to be elucidated; however, a growing body of evidence suggests that activity of these agents might be restricted to a well-defined patient population selected by biomarker strategies to enrich for patients with sensitivity to MET-targeting agents [17]. This could encompass selection of patients with the highest MET expression and/or *c-met* amplification.

In our study, we observed a statistically significant association between *c-met* amplification and high MET expression, with three patients harboring a *c-met* amplification among the four patients with the highest MET expression (*H*-score ≥ 210). This finding suggests that both FISH and IHC might be appropriate methods for identifying a similar patient population depending on the cut point used. However, exploratory biomarker analyses assessing different dichotomous cut points for MET expression did not reveal any clear statistically significant relationship between MET expression and clinical outcomes. In part, this may be because of the small sample size of 15 patients in this study, but could also reflect the need for additional or complementary biomarkers for patient selection.

According to the literature, the activity of MET-targeting agents might be restricted to patients in whom MET is the sole driver of the disease [17]. As downstream signaling pathways common to MET and the human epidermal growth factor receptor (HER) family partially overlap, it has been hypothesized that the effect of MET inhibition could be neutralized or attenuated by parallel activation of HER family receptors, such as epidermal growth factor receptor [18]. In our study, there were interesting anecdotal observations in line with this hypothesis for some patients. Out of the three patients enrolled in our study who were harboring *c-met* amplification and corresponding high MET protein expression (*H*-score ≥ 210), two did not show any reduction in target lesions following emibetuzumab treatment (Fig. 2, patients 1 and 3), and both were diagnosed as having tumors with co-amplification of *EGFR*. In contrast, the only patient with *c-met* amplification, but no *EGFR* amplification, was the patient with the greatest reduction in target lesions in our study (Fig. 2, Patient 15). Despite a − 22.4% change in tumor size from baseline, the patient was discontinued at the end of Cycle 2 because of PD based on a new liver lesion. This mixed response suggests that the target lesions, at least, in this patient might have been MET-dependent and sensitive to MET-targeted emibetuzumab therapy as hypothesized for MET-amplified tumors without concomitant *EGFR* signaling.

Concomitant *c-met* and *EGFR* amplification and activation of extensive crosstalk between HER and MET receptors have been reported to be sources of drug resistance in gastric cancer [19, 20]. Amplification of *c-met* is observed in 4–12% of gastric cancer patients and concomitant *c-met* and *EGFR* amplification (or overexpression of the respective proteins) has been reported in up to 6% of gastric cancer cases [21, 22]. Therapeutic strategies that have been suggested as promising options for patients with high MET expression or *c-met* amplification, to overcome primary and acquired drug resistance, include combination therapies that target MET and EGFR, as well as therapies utilizing tetraspecific antibodies [18–20]. For example, monotherapy with MET inhibitors was found to induce tumor growth inhibition only in a cohort of patient-derived xenografts from a poorly differentiated adenocarcinoma of the GEJ with *c-met* amplification, but no *EGFR* molecular alterations. In contrast, concomitant MET/EGFR inhibition resulted in complete tumor regression and prevented the onset of resistance in this cohort of patient-derived xenografts with *c-met* amplification, but no *EGFR* molecular alterations [18].

In addition, combination of standard of care chemotherapy regimens and antibodies targeting MET signaling have been studied. However, the results of late stage trials for the anti-HGF antibody rilotumumab and the monovalent anti-MET antibody onartuzumab in combination with chemotherapy did not show a survival benefit [17, 23, 24]. There are several possible explanations for the negative results obtained with these antibodies (which, in contrast to emibetuzumab, only block ligand-dependent but not ligand-independent MET signaling), including failure to identify the appropriate target population for these compounds [17].

Emibetuzumab had a favorable safety profile in our study as in the previous studies and there were no new unexpected safety findings. The reported emibetuzumab-related AEs were mostly mild or moderate in intensity and the most common were hypoalbuminemia (a known class effect for agents targeting MET or HGF) [25–27] and constipation. Only three emibetuzumab-related Grade ≥ 3 AEs were reported (hyperkalemia, hyponatremia, and hyperuricemia). No thromboembolic events were observed, whereas these events have been reported for the monovalent anti-MET antibody onartuzumab [28].

The pharmacokinetic profile of emibetuzumab in this Asian study population was similar to the profiles observed in the previous studies of emibetuzumab monotherapy, which were conducted in the United States with mostly Caucasian patients and supported a 2-week dosing schedule [29, 30]. Analysis of the pharmacokinetic data from this study is underway and the data will be combined with data from other studies in a population pharmacokinetics report.

One limitation of this report is the small sample size, and the non-randomized, single-arm study design, which has known inferential deficiencies. However, the study design was appropriate for assessing the primary endpoint of PFS rate at 8 weeks in a limited number of patients to explore putative evidence of antitumor activity in the selected patient population.

In conclusion, although the study did not meet its primary endpoint, emibetuzumab was well tolerated and showed limited single-agent activity in Asian patients with advanced gastric adenocarcinoma. Further investigation, including complementary biomarkers to identify patients with gastric cancer who are most likely to benefit from emibetuzumab single-agent therapy, is warranted. Given the favorable safety profile, combination strategies with other therapeutic agents in patients with MET-positive gastric cancer appear feasible and might provide strategies for improving the activity of emibetuzumab in this patient population in the future.
